# Cigarette Smoking, Reduction and Quit Attempts: Prevalence Among Veterans With Coronary Heart Disease

**DOI:** 10.5888/pcd13.150282

**Published:** 2016-03-24

**Authors:** Troy A. Shahoumian, Barbara R. Phillips, Lisa I. Backus

**Affiliations:** Author Affiliations: All authors were affiliated with the Population Health Program, Office of Public Health, Veterans Health Administration, Palo Alto, California. Dr Phillips recently retired.

## Abstract

**Introduction:**

Cigarette smoking increases the risk of illness and early death for people with coronary heart disease. In 2010, Brown estimated prevalence rates for smoking among veterans and nonveterans with or without coronary heart disease in the United States, based on the 2003 through 2007 data from the Behavioral Risk Factor Surveillance System (BRFSS). Recent changes in BRFSS methods promise more accurate estimates for veterans. To inform assessment of efforts to reduce smoking, we sought to provide prevalence rates for smoking behaviors among US veterans with coronary heart disease and to compare rates for veterans with those for civilians.

**Methods:**

We conducted a cross-sectional analysis of participants who responded to BRFSS from 2009 to 2012. Accounting for complex BRFSS sampling, we estimated national prevalence rates by sex for smoking status, frequency, and quit attempts; for those with and those without coronary heart disease; for civilians; for veterans and active duty personnel combined; and, after adjusting for BRFSS mingling of active duty personnel and veterans, for veterans only. We examined differences between veterans and civilians by using age-standardized national estimates.

**Results:**

Among men with coronary heart disease, more veterans than civilians smoked and more were daily smokers, but veterans were no more likely to attempt to quit. Among women with coronary heart disease, we found no differences between civilians and veterans.

**Conclusion:**

Cigarette smoking is more prevalent among male veterans with coronary heart disease than among their civilian counterparts. Not distinguishing active duty personnel from veterans can materially affect prevalence estimates intended to apply solely to veterans.

## Introduction

Cigarette smoking puts people with coronary heart disease at increased risk of illness and death (1,2). Fortunately, smoking cessation reduces this risk rapidly and markedly (3). Reduction in smoking predicts eventual cessation among several groups (4–8). Nonetheless, smoking cessation is complex and dynamic; smokers typically make multiple transitions between smoking, reduction in smoking, and cessation (9–12).

Brown reported age-standardized prevalence rates for current cigarette smoking in the United States among veterans and nonveterans with coronary heart disease and those without it (13). His analysis drew on data from the Behavioral Risk Factor Surveillance System (BRFSS) for 2003 through 2007. To our knowledge, BRFSS is the only large, ongoing national survey that collects information both on veteran status and diagnosis of coronary heart disease. Before the current work, the Brown study was the only one to address smoking prevalence among US veterans with coronary heart disease. Our analysis extends Brown’s work; Brown addressed only current smoking and did not test the statistical significance of differences between veterans and civilians. Mindful of the importance of smoking to public health, we sought to inform assessment of efforts to reduce smoking among veterans and among those with coronary heart disease using more recent BRFSS data. We compare prevalence of cigarette smoking status, smoking frequency, and attempt to quit smoking, by sex, in the United States among veterans and civilians with and without coronary heart disease.

## Methods

The primary data sources for this analysis were BRFSS individual-level public use files for 2011 and 2012. We used individual-level BRFSS data for 2009 and 2010 to adjust our estimates for 2011 and 2012. We pooled data for 2011 and 2012 and for 2009 and 2010.

BRFSS monitors prevalence of major behavioral health risks among the noninstitutionalized adult population of the United States using a voluntary, annual, cross-sectional, random-digit–dialed telephone survey (14). The sample design is state-based; all 50 states, the District of Columbia, and major US territories administer the survey. In 2011–2012, more than 980,000 BRFSS interviews were completed, including more than 124,000 with those who had served in the US Armed Forces.

The complex sample design and weighting of BRFSS necessitates specialized analytic procedures. To pool data across 2 years, we generated unique strata by year and applied weights for the relevant year. We used SAS SurveyMeans and SurveyReg procedures (version 9.2, SAS Institute) to ensure correct computation of variance and the SAS DOMAIN command to specify subgroups of the population. We calculated two-tailed 95% confidence intervals. Reading the overlap of confidence intervals yields only approximate *P* values (15). For this reason, we also calculated 95% *F* statistics to formally test differences.

The BRFSS questions used in this analysis are available online (16). Like Brown, we considered a respondent to have coronary heart disease if he or she reported a diagnosis of heart attack, angina, or coronary heart disease by a health care provider (13). We used the BRFSS measure of smoking status, which classified those who reported smoking at least 100 cigarettes as current smokers or former smokers, depending on whether they reported smoking cigarettes during the past 30 days. Brown used the same BRFSS measure to identify current smokers. Following BRFSS, we defined those who reported smoking fewer than 100 cigarettes in their lives as having never smoked. We used the BRFSS measure of frequency of cigarette smoking, which distinguished between smoking every day and only some days during the past 30 days. We also used the BRFSS measure of a quit attempt, which asked current smokers whether they had not smoked for at least 1 day in an attempt to quit during the previous 12 months.

We defined a veteran as someone no longer in the Armed Forces of the United States. This definition is consistent with the federal definition and with the basic criteria for receiving benefits from the US Department of Veterans Affairs (VA); those currently on active duty are not eligible for VA benefits. We defined a civilian as someone who never served on active duty in the US Armed Forces.

From 2009 through 2012, the BRFSS question ascertaining military service was worded identically; it asked, “Have you ever served on active duty in the United States Armed Forces, either in the regular military or in a National Guard or military reserve unit?” However, the response categories changed during this period. In 2011 and 2012, the responses to this question did not distinguish current service (active duty personnel) and past service (veterans). From 2003 through 2007 — the period that Brown analyzed — BRFSS also did not distinguish current and past service (13,16). The inability to distinguish active duty personnel from veterans is problematic because the 2 groups may differ appreciably, potentially leading to biased results if active duty personnel are treated as veterans. In contrast, in 2009 and 2010, the BRFSS response categories did distinguish veterans from active duty personnel (16).

Given the available data, we estimated 2011–2012 prevalence rates (point estimates and confidence intervals) for veterans and active duty personnel combined and we formally tested the differences between the rates for civilians and for veterans and active duty personnel combined. We used 2009–2010 data to adjust the 2011–2012 point estimates to exclude the effect of active duty personnel, thus deriving adjusted point estimates of prevalence rates for veterans.

To determine the adjustment, we estimated 2009–2010 prevalence rates twice — with active duty personnel first excluded and then included; the ratio between these rates is our estimate of the effect of the inclusion of active duty personnel. We assumed this effect was the same in 2011–2012 as in 2009–2010. For example, if the ratio between the 2009 and 2010 rate excluding active duty personnel and the 2009–2010 rate including such personnel was 0.95, we assumed that the comparable ratio for 2011–2012 rate was also 0.95; and we reduced the 2011–2012 estimate including active duty personnel by multiplying it by 0.95.

We assessed the sensitivity of the 2011–2012 unadjusted results to the inclusion of active duty personnel by comparing the adjusted and unadjusted estimates. For simplicity, we rounded both the unadjusted and adjusted rates to the nearest whole percentage point before comparing them. We report adjusted rates only if they differed (after rounding) from the unadjusted rates. Because the adjustment was based on an aggregate ratio, rather than individual-level data, it is not possible to directly assess the statistical significance of a difference involving an adjusted rate. We treat an adjusted difference as statistically significant only if 1) the corresponding, unadjusted difference was statistically significant and 2) the magnitude of the adjusted difference was no smaller than that of the unadjusted difference.

Starting with the 2011 survey, BRFSS methodology fundamentally changed. Among other changes, a sampling frame based on cellular telephone numbers was added to represent better the growing segment of the US population that does not have a landline. Estimates of smoking prevalence based only on landline surveys are biased (17).

Because the age distribution of veterans does not match the age distribution of civilians, one must control for age when comparing these 2 groups in formal tests of differences between civilians and a combination of veterans and active duty personnel, and (for the sensitivity analysis) between civilians and veterans. To control for age, our primary analysis uses age-standardized estimates that are based on the US adult population in 2010–2012 (18). Identical age bands were constructed by sex for veterans and active duty personnel combined, for veterans alone, and for civilians. Because of the limited number of observations for younger veterans, we grouped people aged 18 to 34 years into 1 age band and those aged 35 to 44 into another. Older people were grouped into 10 five-year age bands (up to age 80). In addition, we estimated prevalence rates and computed formal tests of differences between veterans and active duty personnel combined and for civilians in each of the 10 age bands. This age-band analysis supports assessment of patterns of prevalence across different age groups.

The participants in this study were BRFSS respondents from 2009 through 2012. Veterans, active duty personnel, and civilians who responded in 2011–2012 were the primary participants. Veterans and active duty personnel who responded in 2009 and 2010 were participants in the sensitivity analysis.

The public use files of the BRFSS contain no personal identifiers. We did not seek human subject approval because it is not necessary in these circumstances.

## Results

### Descriptive analysis

Descriptive estimates of the smoking behavior of the populations of interest (veterans and active duty personnel, veterans and civilians) appear below and are not age standardized.

Coronary heart disease was not uncommon among women in 2011–2012. We estimated that 5.2% (95% CI, 4.4%–6.1%) of female veterans and active duty personnel had coronary heart disease in 2011–2012. After adjustment to exclude active duty personnel and rounding (hereafter adjustment), the point estimate for veterans is 6%. Among female civilians, 5.3% (95% CI, 5.2%–5.4%) had coronary heart disease in 2011–2012.

We estimated that 27.9% (95% CI, 21.7%–34.1%) of female veterans and active duty personnel with coronary heart disease currently smoked cigarettes in 2011–2012; after adjustment, the estimate for veterans was 27%. We estimated that 19.7% (95% CI, 14.4%–25.1%) of female veterans and active duty personnel with coronary heart disease smoked every day; after adjustment, the estimate for veterans was 18%. Thus, daily smokers are roughly two-thirds of female veterans with coronary heart disease who smoke. About 7 in 10 (69.6%; 95% CI, 59.2%–80.1%) female veterans and active duty personnel with coronary heart disease who smoked had attempted to quit during the previous 12 months (this estimate was unchanged by adjustment). We estimated that 31.2% (95% CI, 24.8%–37.5%) of female veterans and active duty personnel with coronary heart disease were former cigarette smokers in 2011–2012. After adjustment, the estimate for veterans was 32%. About 4 in 10 (41.0%; 95% CI, 32.0%–49.9%) female veterans and active duty personnel with coronary heart disease never smoked (unchanged by adjustment).

Among civilian women with coronary heart disease, we estimated that 20.5% (95% CI, 19.6%–21.3%) were current smokers and that 14.7% (95% CI, 13.9%–15.4%) smoked every day. Among current smokers, 68.3% (95% CI, 66.2%–70.5%) had a quit attempt. About a third (32.7%; 95% CI, 31.9%–33.6%) of civilian women with coronary heart disease were former smokers. Nearly half (46.8%; 95% CI, 45.8%–47.8%) of female civilians with coronary heart disease never smoked.

Coronary heart disease was relatively common among male veterans and active duty personnel in 2011–2012. We estimated that 16.5% (95% CI, 16.1%–16.9%) of male veterans and active duty personnel had coronary heart disease in 2011–2012. The comparable rate for male civilians was 5.9% (95% CI, 5.8%–6.1%).

We estimated that 16.9% (95% CI, 15.9%–17.9%) of male veterans and active duty personnel with coronary heart disease smoked cigarettes in 2011–2012 (unchanged by adjustment) and that 12.7% (95% CI, 11.8%–13.6%) smoked every day (unchanged by adjustment). Thus, daily smokers are about three quarters of male veterans with coronary heart disease who smoke. Approximately 6 in 10 (57.0%; 95% CI, 53.7%–60.3%) male veterans and active duty personnel with coronary heart disease who smoked had a quit attempt (unchanged by adjustment). We estimated that 59.2% (95% CI, 58.0%–60.4%) of male veterans and active duty personnel with coronary heart disease were former cigarette smokers in 2011–2012 (unchanged by adjustment). Less than a quarter (23.9%; 95% CI, 22.9%–24.9%) of male veterans and active duty personnel with coronary heart disease never smoked. After adjustment, the estimate for veterans was 23%.

Among male civilians with coronary heart disease, we estimated that 24.5% (95% CI, 23.4%–25.7%) were current smokers and 17.3% (95% CI, 16.3%–18.3%) of such civilians smoked every day. Among these current smokers, 63.0% (95% CI, 60.3%–65.8%) had a quit attempt. Approximately 4 in 10 (42.7%, 95% CI, 41.5%–44.0%) male civilians with coronary heart disease were former smokers. About a third (32.7%; 95% CI, 31.4%–34.0%) of male civilians with coronary heart disease never smoked.

### Age-standardized results

We present primarily age-standardized estimates; these are supplemented with discussion of the age-band analysis. The age-standardized estimates are for veteran and active duty personnel and civilians, along with adjusted estimates for veterans only. The age-band estimates appear online in Appendix Tables 1–3.

Among those with coronary heart disease, we found differences between veteran and active duty personnel and civilians in smoking status and frequency for men, but not for women. Current smoking among male veterans and active duty personnel with coronary heart disease was more prevalent than among their civilian counterparts; the magnitude of the difference was large and increased with adjustment to exclude active duty personnel (Table 1). This difference in the prevalence of current smoking among men with coronary heart disease was accompanied by a difference between veterans and active duty personnel and civilians in the prevalence of smoking every day rather than only some days, which was also large in magnitude and increased with adjustment (Table 2). Male veterans and active duty personnel with coronary heart disease were less likely than their civilian counterparts to have never smoked; the magnitude of this difference was large and increased with adjustment (Table 1).

**Table 1:** Cigarette Smoking Status^a^ Among Adults With and Without Coronary Heart Disease by Sex and Veteran Status — United States, 2011–2012

Disease Status by Sex and Smoking Status	Prevalence Rate (95% CI)		Difference in Prevalence Rate* (P* Value)^b^^,^ c
	Veterans and Active Duty Personnel	Civilians	
**With coronary heart disease**			
Women (775 veterans and active duty personnel; 42,573 civilians)			
Current smoker	32.3 (22.5–42.1)^d^	29.6 (27.7–31.6)	2.7 (.60)
Former smoker	26.7 (18.7–34.8)	23.8 (22.2–25.4)	2.9 (.49)
Never smoker	41.0 (28.5–53.4)^e^	46.5 (44.4–48.7)	−5.6 (.39)
Men (21,841 veterans and active duty; 22,824 civilians)			
Current smoker	43.5 (35.8–51.2)^f^	32.4 (30.0–34.8)	11.1 (.01)
Former smoker	31.9 (26.5–37.4)	31.4 (29.3–33.4)	0.6 (.85)
Never smoker	24.6 (17.5–31.6)^g^	36.2 (33.6–38.8)	−11.7 (.002)
**Without coronary heart disease**			
Women (9,347 veterans and active duty personnel; 520,280 civilians)			
Current smoker	19.1 (17.6–20.6)^h^	16.7 (16.5–16.9)	2.4 (.002)
Former smoker	27.3 (25.7–28.8)	20.7 (20.5–20.9)	6.6 (<.001)
Never smoker	53.7 (51.8–55.5)^i^	62.6 (62.4–62.9)	−9.0 (<.001)
Men (88,895 veterans and active duty; 245,625 civilians)			
Current smoker	24.0 (23.2–24.8)^j^	20.9 (20.6–21.3)	3.1 (<.001)
Former smoker	32.5 (31.8–33.2)	25.9 (25.6–26.3)	6.6 (<.001)
Never smoker	43.5 (42.6–44.4)^k^	53.1 (52.7–53.5)	−9.7 (<.001)

Source: Behavioral Risk Factor Surveillance System, 2011 and 2012, with adjustments based on data from 2009 and 2010.

Abbreviation: CI, confidence interval.

a Smokers reported smoking at least 100 cigarettes in their lifetimes; current smokers reported smoking during past 30 days. Estimates are age-standardized.

b Due to rounding, the difference presented in this column may differ from that the result obtained by subtracting the rate for civilians from the rate for veterans and active duty personnel.

c *P* value of difference in rates of veterans and active duty and civilians, obtained from an *F* test.

d After adjustment to exclude active duty personnel, the point estimate is 30%.

e After adjustment to exclude active duty personnel, the point estimate is 43%.

f After adjustment to exclude active duty personnel, the point estimate is 45%.

g After adjustment to exclude active duty personnel, the point estimate is 23%.

h After adjustment to exclude active duty personnel, the point estimate is 20%.

i After adjustment to exclude active duty personnel, the point estimate is 53%.

j After adjustment to exclude active duty personnel, the point estimate is 25%.

k After adjustment to exclude active duty personnel, the point estimate is 42%.

**Table 2:** **Frequency of Cigarette Smoking Among Adults^a^ With and Without Coronary Heart Disease by Sex and Veteran Status** — **United States, 2011–2012**

Disease Status by Sex and Smoking Frequency	Prevalence Rate (95% CI)		Difference in Prevalence Rate* (P* Value)^b^^,^ c
	Veterans and Active Duty Personnel	Civilians	
**With coronary heart disease**			
Women (775 veterans and active duty; 42,573 civilians)			
Every day	22.8 (14.5–31.2)^d^	22.3 (20.5–24.1)	0.6 (.89)
Some days	9.5 (4.7–14.3)	7.3 (6.3–8.4)	2.1 (.40)
Men (21,841 veterans and active duty; 22,824 civilians)			
Every day	32.4 (24.9–39.9)^e^	22.3 (20.3–24.4)	10.1 (.01)
Some days	11.1 (6.6–15.7)^f^	10.1 (8.4–11.8)	1.0 (.68)
**Without coronary heart disease**			
Women (9,347 veterans and active duty; 520,280 civilians)			
Every day	14.2 (12.9–15.6)^g^	12.0 (11.8–12.2)	2.3 (.001)
Some days	4.8 (4.1–5.6)	4.7 (4.6–4.8)	0.1 (.77)
Men (88,895 veterans and active duty; 245,625 and civilians)			
Every day	17.4 (16.7–18.1)^h^	14.5 (14.2–14.7)	2.9 (<.001)
Some days	6.7 (6.2–7.2)	6.5 (6.3–6.7)	0.2 (.51)

Source: Behavioral Risk Factor Surveillance System, 2011 and 2012 primarily, with adjustments based on data from 2009 and 2010.

Abbreviation: CI, confidence interval.

a Frequency of cigarette smoking as reported during the past 30 days.

b Because of rounding, the difference presented in this column may differ from that the result obtained by subtracting the rate for civilians from the rate for veterans.

c *P* value of difference in rates of veterans and civilians, obtained from an *F* test.

d After adjustment to exclude active duty personnel, the point estimate is 21%.

e After adjustment to exclude active duty personnel, the point estimate is 33%.

f After adjustment to exclude active duty personnel, the point estimate is 12%.

g After adjustment to exclude active duty personnel, the point estimate is 15%.

h After adjustment to exclude active duty personnel, the point estimate is 18%.

Among current smokers with coronary heart disease, we found no difference in the prevalence of a quit attempt after adjustment between veterans and civilians of either sex (Table 3). Among women, the difference in the prevalence of a quit attempt between veterans and active duty personnel and civilians was of substantial magnitude (7.7 percentage points) and significant. However, this magnitude was much diminished after adjustment, leaving only a 1 percentage point difference between veterans and civilians.

**Table 3:** **Attempt to Quit Smoking Cigarettes Among Adult Current Smokers^a^ With and Without Coronary Heart Disease by Sex and Veteran Status** — **United States, 2011–2012^b^**

Disease Status by Sex	Veterans and Active Duty		Civilians		Veterans or Active Duty Personnel vs Civilians, Difference (*P* Value)^c^
	No. of Observations	Prevalence Rate (95% CI)	No. of Observations	Prevalence Rate (95% CI)	
**With coronary heart disease**					
Women	200	75.0 (68.4–81.7)^d^	7,436	67.4 (63.9–70.9)	7.7 (.04)
Men	3,219	60.5 (51.1–69.9)	4,696	63.7 (59.9–67.5)	−3.2 (.54)
**Without coronary heart disease**					
Women	1,823	57.8 (53.6–62.0)	78,612	58.9 (58.2–59.5)	1.1 (.62)
Men	14,676	55.9 (54.3–57.6)	47,039	57.3 (56.3–58.3)	1.3 (.18)

Source: Behavioral Risk Factor Surveillance System, 2011 and 2012 primarily, with adjustments based on 2009 and 2010.

Abbreviation: CI, confidence interval.

a Current smokers who reported stopping smoking for at least 1 day in an attempt to quit during the past 12 months are defined as having a quit attempt.

b Estimates are age-standardized.

c Because of rounding, the difference presented in this column may differ from that the result obtained by subtracting the rate for civilians from the rate for veterans.

d After adjustment to exclude active duty personnel, the point estimate is 69%.

Among both women and men without coronary heart disease, we found differences between veterans and active duty personnel and civilians in smoking status and frequency, but not quit attempts. Among both sexes, fewer veterans and active duty personnel without coronary heart disease than their civilian counterparts had never smoked and fewer were former smokers; with adjustment, the differences between veterans and civilians increased slightly or were unchanged (Table 1). Higher rates of cessation among veterans and active duty personnel did not fully offset higher lifetime smoking rates; among each sex, more veterans and active duty personnel than civilians of each sex were current smokers; after adjustment, the difference between civilians and veterans increased slightly (Table 1).

We also estimated that both male and female veterans and active duty personnel without coronary heart disease were daily smokers than their civilian counterparts; after adjustment, the difference between civilians and veterans increased slightly (Table 2).

**Results by age band**. Among women we identified few clear patterns of significant differences across age bands between veterans and active duty personnel and civilians (Appendix Tables 1–3). Among women with coronary heart disease, we identified no such patterns. Among women without coronary heart disease, we identified only 1: female veterans and active duty personnel in each age band were less likely to be never smokers than their civilian counterparts.

Among men, we identified multiple clear patterns of differences between veterans and active duty personnel and civilians across age bands both for those with and for those without coronary heart disease (Appendix Tables 1–3). Male veterans and active duty personnel with or without coronary heart disease had a higher prevalence of current smoking and of daily smoking than their civilian counterparts, and male veterans and active duty personnel without coronary heart disease had a lower prevalence of never having smoked and a higher prevalence of being former smokers than their civilian counterparts. We observed an interesting pattern of differences in the likelihood of former smoking across age bands among men with coronary heart disease: among such men aged 50 years or older, veterans and active duty personnel were *more* likely to be former smokers than their civilian counterparts, but among those aged 49 years and younger, veterans and active duty personnel were *less* likely to be former smokers. Recall that in the age-standardized analysis we found no difference between male veterans and active duty personnel with coronary heart disease and their civilian counterparts in the prevalence of being a former smoker. In this case, the age-standardized result apparently obscured disparate smoking behaviors across age bands.

**Effect of inclusion of active duty personnel.** We found that the effect on prevalence rates of mingling active duty personnel and veterans was not large, but it was occasionally material in that it altered the interpretation of results. Among veterans with coronary heart disease, the adjusted rate ranged from 92% to 110% of the unadjusted rate for women and from 92% to 109% for men. The ranges were somewhat narrower for veterans without coronary heart disease. Nevertheless, the magnitude of the difference between veterans and active duty personnel and civilians occasionally was so diminished by adjustment for the inclusion of active duty personnel that the small difference that remained between veterans and civilians was likely not clinically meaningful, thus altering the interpretation of results.

## Discussion

For male and female veterans without coronary heart disease and for male veterans with coronary heart disease, our finding that prevalence rates for smoking during one’s lifetime are higher among veterans than among their civilian counterparts is consistent with other evidence. Rates of cigarette smoking have been higher historically among veterans than among people in the general population (19).

Our estimates of age-standardized prevalence rates for current smoking among those with coronary heart disease are broadly consistent with those of Brown (2010), despite the different periods covered, changes in BRFSS methodology, and our adjustment for inclusion of active duty personnel (13). For veterans, for example, estimated prevalence rates for current smoking (after adjustment) were up to 5% higher than Brown’s estimates for those with coronary heart disease and up to 13% lower for those without coronary heart disease. It is not possible to determine to what extent the differences between our estimates and those of Brown are due to actual changes in smoking prevalence in the populations of interest or to differences in methods and measures, including the addition of a cellular telephone sample to BRFSS and more accurate identification of rates for veterans.

One limitation of this analysis is that not all veterans, as defined here, are eligible for VA benefits. Most VA benefit programs exclude former service members who were dishonorably discharged; other eligibility requirements vary from one VA program to another. No BRFSS data are available on type of discharge or on any other eligibility criteria for VA benefits.

Adjustment to exclude active duty personnel is a strength of the current analysis, especially given that the characteristics of active duty personnel and those of veterans are unlikely to have changed appreciably between 2009–2010 and 2011–2012. Nonetheless, this adjustment has limitations. The data used in the adjustment were collected before major changes to BRFSS methodology in 2011, including the addition of the cellular telephone sample. For the adjustment, we assumed that the ratios of estimates excluding and including active duty personnel were the same in 2011–2012 as in 2009–2010. The available data do not permit an assessment of the extent to which differences in BRFSS methodology may invalidate that assumption. Another limitation related to the adjustment is that tests of statistical significance must rely on the unadjusted estimates. The BRFSS question on service in the US Armed Forces needs to be revised so that active duty personnel readily can be distinguished from veterans in future analyses of BRFSS data.

Another limitation of this analysis is that the number of observations on young veterans and active duty personnel with coronary heart disease is limited. The limited number of observations for younger age bands, especially among women, could introduce error into our age-adjusted estimates because the available observations may poorly represent young veterans and active duty personnel generally. Our estimates for each of the 10 separate age bands are useful in assessing the extent of possible error due to limited numbers of observations in some age bands. As indicated above, the age-band estimates for women with coronary heart disease do not show clear patterns, suggesting that the age-adjusted estimates for such women should be interpreted with caution. Among men with coronary heart disease, on the other hand, the pattern of differences across age bands between veterans and active duty personnel and civilians generally tracks the pattern of age-adjusted differences between the 2 bands. The single exception apparently is not due to erratic results but rather to a curvilinear relationship across age bands. We obtained generally consistent results using 2 techniques to control for age, which suggests that our estimates for men with coronary heart disease are robust.

This analysis is based on survey data, which have inherent limitations that may affect our estimated prevalence rates. First, response rates were lower than desirable in many states and territories participating in the BRFSS (20). However, BRFSS applies statistical techniques to correct for nonresponse, and multiple studies have concluded that BRFSS provides valid national estimates (21,22). Second, all the data used are based on self-report, including diagnosis of coronary heart disease and smoking behavior. The evidence on the accuracy of self-report of heart disease is mixed. Based on comparison with hospital records, self-reports of ischemic heart disease leading to hospitalization have been found accurate (23). Also, researchers found substantial agreement between hospital records and self-report of myocardial infarction (24). On the other hand, self-reported myocardial infarction was found to be inconsistent with electrocardiogram records (25).

Although smoking status is routinely measured through self-report, these measures are sometimes questioned under the assumption that smokers tend to underestimate the amount smoked or even deny smoking. Roughly 2 decades ago, a meta-analysis on validity of self-reported smoking found wide variation in reporting of smoking status but concluded that self-reports were accurate in most studies (26). These authors also concluded that interviewer-administered questionnaires and reports by adults — both of which characterize BRFSS data — were associated with greater accuracy. A more recent study found that a history of myocardial infarction did not predict invalid reporting of smoking status (27). Furthermore, analysis of a nationally representative sample of adults found only a small discrepancy between urinary cotinine concentrations and self-reported smoking status (28).

In recent years, the VA’s Veterans Health Administration (VHA) has devoted much effort to reducing smoking among those veterans in VHA care (29). Perhaps that effort is at least partly responsible for our finding that, among those without coronary heart disease, all veterans — both male and female — were more likely to be former smokers than all civilians. Receipt of a doctor’s advice to give up smoking was found to be associated with cessation, with a quit attempt, and possibly with reduction in smoking (8,30). However, BRFSS does not measure use of VHA medical care. Therefore, with the available data, we cannot further investigate the VHA’s role in reducing smoking.

Smoking cessation is critical for smokers with coronary heart disease, and clinicians have a responsibility to help patients with coronary heart disease reduce and ultimately quit smoking. For male veterans with coronary heart disease — who are more likely to smoke than their civilian counterparts — the need is particularly great. The rates of quit attempts among current smokers with coronary heart disease for both veterans and civilians are encouraging and suggest that patients and providers are aware of the need to quit. The rates of current smoking, however, suggest that more effective interventions may be necessary for veterans with coronary heart disease so that motivation to quit results in successful smoking cessation.

## Appendix

Supplemental Tables 1–3. This file is available for download as a Microsoft Word document.
