# Supplementary material for: Cigarette Smoking, Reduction and Quit Attempts: Prevalence Among Veterans With Coronary Heart Disease
Source: Prev Chronic Dis. 2016 Mar 24;13:E41. doi: 10.5888/pcd13.150282 (PMC4807437; doi:10.5888/pcd13.150282)
Supplement: Supplementary file 1 [file 15_0282_Appendix.docx]

Table 1. Cigarette Smoking Status Among Adults With and Adults Without Coronary Heart Disease by Sex, Age Group, and Veteran Status — United States, 2011–2012

| Disease Status by Sex, Age Group, and Veteran Status | Prevalence Rate, % (95% Confidence Interval) | | Difference Between Veterans/Active Duty Personnel and Civilians^a^ (*P* Value)^b^ |
| --- | --- | --- | --- |
|  | Veterans or Active Duty Personnel | Civilians |  |
| **With Coronary Heart Disease** | | | |
| **Women** | | | |
| Aged 18–34 years (veterans or active duty personnel: 23; civilians: 581) | | | |
| Current smoker | 27.7 (0.0–55.6) | 29.0 (23.4–34.6) | −1.3 (.07) |
| Former smoker | 16.8 (0.0–37.6) | 14.0 (9.5–18.5) | 2.8 (.01) |
| Never smoker | 55.5 (16.7–94.4) | 57.0 (50.7–63.4) | −1.5 (.001) |
| Aged 35–44 years (veterans or active duty personnel: 32; civilians: 1,278) | | | |
| Current smoker | 43.7 (19.8–67.6) | 39.9 (34.8–44.9) | 3.8 (.31) |
| Former smoker | 30.0 (5.4–54.7) | 17.5 (13.6–21.4) | 12.5 (.03) |
| Never smoked | 26.3 (8.5–44.0) | 42.6 (37.6–47.7) | −16.4 (.01) |
| Aged 45–49 years (veterans or active duty personnel: 38; civilians: 1,425) | | | |
| Current smoker | 29.6 (6.3–52.8) | 43.1 (37.8–48.3) | −13.5 (.72) |
| Former smoker | 16.0 (0.0–36.2) | 19.5 (15.8–23.3) | −3.5 (.01) |
| Never smoker | 54.4 (23.9–84.9) | 37.4 (31.7–43.1) | 17.0 (.01) |
| Aged 50–54 years (veterans or active duty personnel: 79; civilians: 2,608) | | | |
| Current smoker | 46.7 (30.2–63.2) | 38.4 (34.9–41.9) | 8.3 (.09) |
| Former smoker | 28.5 (13.8–43.2) | 27.4 (24.0–30.8) | 1.1 (.13) |
| Never smoker | 24.7 (10.1–39.4) | 34.2 (30.8–37.6) | −9.4 (.01) |
| Aged 55–59 years (veterans or active duty personnel: 82; civilians: 3,869) | | | |
| Current smoker | 47.2 (30.0–64.4) | 34.0 (30.9–37.1) | 13.2 (.02) |
| Former smoker | 27.0 (14.2–39.8) | 28.9 (26.2–31.6) | −1.9 (.22) |
| Never | 25.8 (12.4–39.2) | 37.1 (34.1–40.2) | −11.4 (.003) |
| Aged 60–64 years (veterans or active duty personnel: 96; civilians: 5,034) | | | |
| Current smoker | 28.0 (13.5–42.5) | 23.7 (21.5–26.0) | 4.3 (.95) |
| Former smoker | 37.1 (22.0–52.2) | 34.2 (31.7–36.6) | 2.9 (.004) |
| Never smoker | 34.9 (20.5–49.3) | 42.1 (39.5–44.6) | −7.2 (.01) |
| Aged 65–69 years (veterans or active duty personnel: 79; civilians: 5,610) | | | |
| Current smoker | 37.9 (19.7–56.0) | 18.3 (16.4–20.2) | 19.6 (.86) |
| Former smoker | 31.6 (16.6–46.6) | 43.2 (40.6–45.7) | −11.6 (.004) |
| Never smoker | 30.5 (14.9–46.2) | 38.5 (36.1–41.0) | −8.0 (.003) |
| Aged 70–74 years (veterans or active duty personnel: 98; civilians: 5,922) | | | |
| Current smoker | 14.8 (5.5–24.2) | 13.8 (11.2–16.4) | 1.0 (.06) |
| Former smoker | 47.9 (31.5–64.2) | 41.7 (39.1–44.3) | 6.2 (.001) |
| Never smoker | 37.3 (19.6–55.0) | 44.5 (41.9–47.1) | −7.2 (<.001) |
| Aged 75–79 years (veterans or active duty personnel: 82; civilians: 6.024) | | | |
| Current smoker | 16.3 (4.7–27.9) | 7.3 (6.2–8.4) | 9.0 (.09) |
| Former smoker | 54.2 (36.7–71.8) | 39.9 (37.5–42.3) | 14.3 (.005) |
| Never smoker | 29.4 (12.1−46.8) | 52.8 (50.3−55.3) | −23.4 (<.001) |
| Aged 80 or older (veterans or active duty personnel: 166; civilians: 10.222)) | | | |
| Current smoker | 4.3 (0.6−8.0) | 3.1 (2.5−3.6) | 1.2 (0.29) |
| Former smoker | 32.6 (21.1−44.2) | 32.9 (31.1−34.8) | −0.3 (<.001) |
| Never smoker | 63.1 (51.2−75.0) | 64.0 (62.1−65.9) | −0.1 (<.001) |
| **Men** | | | |
| Aged 18–34 years (veterans or active duty personnel: 52; civilians: 506) | | | |
| Current smoker | 61.5 (39.0−84.0) | 36.8 (30.3−43.4) | 24.6 (.01) |
| Former smoker | 14.7 (0.0−30.7) | 18.3 (13.0−23.7) | −3.6 (<.001) |
| Never smoker | 23.8 (2.8−44.8) | 44.8 (37.7−52.0) | −21.0 (<.001) |
| Aged 35–44 years (veterans or active duty personnel:187; civilians: 992) | | | |
| Current smoker | 51.4 (37.8−65.0) | 43.5 (38.2−48.7) | 8.0 (.32) |
| Former smoker | 20.3 (11.6−29.1) | 23.1 (18.7−27.4) | −2.7 (<.001) |
| Never smoker | 28.2 (17.1−39.4) | 33.5 (28.4−38.6) | −5.2 (<.001) |
| Aged 45–49 years (veterans or active duty personnel: 269; civilians: 1,166) | | | |
| Current smoker | 38.9 (27.7−50.1) | 40.7 (35.3−46.2) | −1.9 (.27) |
| Former smoker | 26.6 (17.3−36.0) | 27.1 (22.6−31.7) | −0.5 (.003) |
| Never smoker | 34.5 (24.0−45.0) | 32.1 (27.2−37.0) | 2.4 (<.001) |
| Aged 50–54 years (veterans or active duty personnel: 587; civilians: 2,207) | | | |
| Current smoker | 42.8 (35.6−49.9) | 35.3 (31.3−39.3) | 7.5 (.001) |
| Former smoker | 36.7 (30.0−43.3) | 35.2 (31.3−39.2) | 1.4 |
| Never smoker |  |  | −8.9 (<.001) |
| Aged 55–59 years (veterans or active duty personnel: 1,160; civilians: 3,423) | | | |
| Current smoker | 39.8 (34.9–44.6) | 30.0 (27.0–33.0) | 9.8 (<.001) |
| Former smoker | 42.6 (37.6–47.6) | 38.7 (35.6–41.8) | 3.9 (<.001) |
| Never smoker | 17.7 (14.1–21.2) | 31.3 (28.4–34.3) | −13.6 (<.001) |
| Aged 60–64 years (veterans or active duty personnel: 2,888; civilians: 3,745) | | | |
| Current smoker | 24.8 (22.1–27.6) | 21.5 (18.8–24.1) | 3.4 (<.001) |
| Former smoker | 53.8 (50.6–57.0) | 44.5 (41.1–47.8) | 9.3 (<.001) |
| Never smoker | 21.4 (18.7–24.0) | 34.1 (30.4–37.7) | −12.7 (<.001) |
| Aged 65–69 years (veterans or active duty personnel: 3,623; civilians: 3,733) | | | |
| Current smoker | 18.2 (16.0–20.3) | 16.1 (14.0–18.1) | 2.1 (.03) |
| Former smoker | 63.2 (60.4–66.0) | 54.4 (51.3–57.5) | 8.8 (<.001) |
| Never smoker | 18.7 (16.4–21.0) | 29.5 (26.7–32.3) | −10.9 (<.001) |
| Aged 70–74 years (veterans or active duty personnel: 3,462; civilians: 3,223 | | | |
| Current smoker | 11.4 (9.6–13.2) | 9.7 (7.7–11.6) | 1.7 (.14) |
| Former smoker | 68.3 (65.7–70.9) | 61.3 (58.2–64.5) | 7.0 (<.001) |
| Never smoker | 20.3 (18.1–22.4) | 29.0 (26.1–31.8) | −8.7 (<.001) |
| Aged 75–79 years (veterans or active duty personnel: 3,756; civilians: 2,056) | | | |
| Current smoker | 8.0 (6.1–9.9) | 6.6 (4.8–8.3) | 1.4 (.49) |
| Former smoker | 66.2 (63.4–68.9) | 58.6 (53.9–63.3) | 7.6 (<.001) |
| Never smoker | 25.8 (23.4–28.2) | 34.8 (30.0–39.6) | −9.0 (<.001) |
| Aged 80 years and older (veterans or active duty personnel: 5,857; civilians: 1,773) | | | |
| Current smoker | 2.6 (2.0–3.2) | 2.0 (1.1–3.0) | 0.6 (.87) |
| Former smoker | 67.0 (64.8–69.1) | 61.4 (57.2–65.6) | 5.6 (<.001) |
| Never smoker | 30.4 (28.3–32.5) | 36.6 (32.4–40.7) | −6.2 (<.001) |
| **Without Coronary Heart Disease** | | | |
| **Women** | | | |
| Aged 18–34 years (veterans or active duty personnel: 1,445; civilians: 77,460) | | | |
| Current smoker | 23.2 (19.5–26.8) | 19.8 (19.3–20.3) | 3.4 (.07) |
| Former smoker | 15.3 (12.4–18.2) | 11.2 (10.9–11.6) | 4.1 (.01) |
| Never smoker | 61.5 (57.3–65.8) | 69.0 (68.4–69.6) | −7.5 (.001) |
| Aged 35–44 years (veterans or active duty personnel: 1,617; civilians: 69,438) | | | |
| Current smoker | 20.4 (16.7–24.0) | 18.4 (17.9–18.9) | 2.0 (.31) |
| Former smoker | 19.7 (16.4–22.9) | 16.2 (15.7–16.7) | 3.5 (.03) |
| Never smoker | 60.0 (55.7–64.3) | 65.4 (64.8–66.1) | −5.5 (.01) |
| Aged 45–49 years (veterans or active duty personnel: 1,016; civilians: 42,141) | | | |
| Current smoker | 21.7 (17.3–26.0) | 20.3 (19.6–21.0) | 1.4 (.72) |
| Former smoker | 26.5 (21.3–31.8) | 19.3 (18.6–20.0) | 7.3 (.01) |
| Never smoker | 51.8 (46.2–57.4) | 60.4 (59.5–61.3) | −8.6 (.01) |
| Aged 50–54 years (veterans or active duty personnel: 1,247; civilians: 53,115) | | | |
| Current smoker | 23.7 (19.8–27.5) | 20.5 (19.9–21.2) | 3.1 (.09) |
| Former smoker | 27.6 (23.1–32.0) | 24.1 (23.4–24.7) | 3.5 (.13) |
| Never smoker | 48.8 (43.9–53.6) | 55.4 (54.6–56.2) | −6.6 (.01) |
| Aged 55–59 years (veterans or active duty personnel: 1,104; civilians: 56,436) | | | |
| Current smoker | 22.1 (17.4–26.7) | 17.1 (16.4–17.7) | 5.0 (.02) |
| Former smoker | 29.8 (24.4–35.2) | 26.2 (25.5–26.9) | 3.6 (.22) |
| Never smoker | 48.1 (42.1–54.1) | 56.8 (55.9–57.6) | −8.7 (.003) |
| Aged 60–64 years (veterans or active duty personnel: 876; civilians: 57,787) | | | |
| Current smoker | 12.8 (9.1–16.6) | 13.6 (13.0–14.1) | −0.7 (.95) |
| Former smoker | 38.3 (32.0–44.6) | 29.4 (28.6–30.1) | 9.0 (.004) |
| Never smoker | 48.8 (42.3–55.4) | 57.1 (56.2–57.9) | −8.2 (.01) |
| Aged 65–69 years (veterans or active duty personnel: 577; civilians: 49,722) | | | |
| Current smoker | 8.8 (6.0–11.5) | 11.8 (11.3–12.3) | −3.0 (.86) |
| Former smoker | 50.3 (42.8–57.8) | 34.4 (33.6–35.2) | 15.9 (.004) |
| Never smoker | 41.0 (33.7–48.2) | 53.8 (52.9–54.7) | −12.9 (.003) |
| Aged 70–75 years (veterans or active duty personnel: 427; civilians: 40,130) | | | |
| Current smoker | 14.1 (8.6–19.5) | 8.8 (8.3–9.3) | 5.3 (.06) |
| Former smoker | 47.0 (39.3–54.6) | 34.6 (33.7–35.5) | 12.3 (.001) |
| Never smoker | 38.9 (31.7–46.1) | 56.6 (55.6–57.5) | −17.6 (<.001) |
| Aged 75–79 years (veterans or active duty personnel: 349; civilians: 31,359) | | | |
| Current smoker | 10.8 (4.4–17.3) | 6.8 (6.3–7.3) | 4.1 (.09) |
| Former smoker | 45.3 (35.8–54.8) | 33.3 (32.4–34.3) | 11.9 (.01) |
| Never smoker | 43.9 (34.2–53.6) | 59.9 (58.9–60.9) | −16.0 (<.001) |
| Aged 80 years or older (veterans or active duty personnel: 689; civilians: 42,692) | | | |
| Current smoker | 4.5 (2.2–6.8) | 3.3 (3.0–3.6) | 1.2 (.29) |
| Former smoker | 46.9 (40.5–53.3) | 29.4 (28.6–30.3) | 17.5 (<.001) |
| Never smoker | 48.6 (42.2–55.0) | 67.2 (66.4–68.1) | −18.7 (<.001) |
| **Men** | | | |
| Aged 18–34 years (veterans or active duty personnel: 5,483; civilians: 57,363) | | | |
| Current smoker | 29.7 (27.7–31.7) | 27.1 (26.4–27.7) | 2.6 (.01) |
| Former smoker | 20.1 (18.5–21.8) | 13.3 (12.8–13.8) | 6.8 (<.001) |
| Never smoker | 50.1 (47.9–52.4) | 59.6 (58.9–60.3) | −9.5 (<.001) |
| Aged 35–44 years (veterans or active duty personnel: 6,615; civilians: 42,650) | | | |
| Current smoker | 23.0 (21.3–24.8) | 22.4 (21.7–23.2) | 0.6 (0.32) |
| Former smoker | 26.5 (24.7–28.4) | 21.2 (20.5–21.9) | 5.3 (<.001) |
| Never smoker | 50.4 (48.3–52.6) | 56.3 (55.5–57.2) | −5.9 (<.001) |
| Aged 45–49 years (veterans or active duty personnel: 4,823; civilians: 25,358) | | | |
| Current smoker | 22.8 (20.8–24.9) | 21.5 (20.5–22.4) | 1.4 (.27) |
| Former smoker | 28.4 (26.1–30.7) | 23.5 (22.5–24.6) | 4.8 (.003) |
| Never smoker | 48.8 (46.3–51.3) | 55.0 (53.8–56.2) | −6.2 (<.001) |
| Aged 50–54 years (veterans or active duty personnel: 5,779; civilians: 30,089) | | | |
| Current smoker | 29.1 (26.9–31.3) | 22.2 (21.3–23.1) | 6.9 (<.001) |
| Former smoker | 32.5 (30.3–34.7) | 27.2 (26.2–28.1) | 5.3 (<.001) |
| Never smoker | 38.4 (36.1–40.8) | 50.6 (49.5–51.7) | −12.2 (<.001) |
| Aged 55–59 years (veterans or active duty personnel: 6,817; civilians: 30,725) | | | |
| Current smoker | 29.8 (27.7–31.9) | 19.5 (18.6–20.4) | 10.2 (<.001) |
| Former smoker | 37.3 (35.2–39.4) | 32.9 (31.9–34.0) | 4.3 (<.001) |
| Never smoker | 32.9 (30.9–35.0) | 47.5 (46.4–48.7) | −14.6 (<.001) |
| Aged 60–64 years (veterans or active duty personnel: 13,605; civilians: 22,977) | | | |
| Current smoker | 20.2 (18.9–21.5) | 14.9 (13.9–15.8) | 5.3 (<.001) |
| Former smoker | 47.9 (46.3–49.4) | 39.0 (37.7–40.3) | 8.9 (<.001) |
| Never smoker | 31.9 (30.5–33.4) | 46.1 (44.8–47.4) | −14.2 (<.001) |
| Aged 65–69 years (veterans or active duty personnel: 13,946; civilians: 15,559) | | | |
| Current smoker | 14.3 (13.3–15.4) | 12.9 (11.8–14.1) | 1.4 (.03) |
| Former smoker | 53.7 (52.2–55.2) | 45.4 (43.8–47.1) | 8.3 (<.001) |
| Never smoker | 31.9 (30.5–33.3) | 41.6 (39.9–43.3) | −9.7 (<.001) |
| Aged 70–74 years (veterans or active duty personnel: 10,275; civilians: 10,890) | | | |
| Current smoker | 10.5 (9.4–11.5) | 9.7 (8.4–10.9) | 0.8 (.14) |
| Former smoker | 60.6 (58.9–62.4) | 51.1 (49.0–53.2) | 9.5 (<.001) |
| Never smoker | 28.9 (27.3–30.6) | 39.3 (37.2–41.3) | −10.3 (<.001) |
| Aged 75–79 years (veterans or active duty personnel: 9133; civilians: 5,625) | | | |
| Current smoker | 7.0 (6.0–8.0) | 6.9 (5.7–8.1) | 0.1 (.49) |
| Former smoker | 60.5 (58.7–62.4) | 50.3 (47.6–53.0) | 10.2 (<.001) |
| Never smoker | 32.5 (30.7–34.3) | 42.8 (40.1–45.5) | −10.3 (<.001) |
| Aged 80 years or older (veterans or active duty personnel: 12,419; civilians: 4,389) | | | |
| Current smoker | 3.8 (3.2–4.4) | 3.8 (2.6–5.1) | −0.0 (.87) |
| Former smoker | 60.4 (58.8–62.0) | 49.4 (46.0–52.8) | 11.0 (<.001) |
| Never smoker | 35.8 (34.2–37.4) | 46.8 (43.4–50.1) | −11.0 (<.001) |

Source: Behavioral Risk Factor Surveillance System, 2011 and 2012.

^a^ Estimates are *not* age standardized within age group. Smokers are defined as those reporting they smoked at least 100 cigarettes in their lifetime; current smokers reported smoking during the previous 30 days.

^b^ Because of rounding, the difference presented in this column may differ from the result obtained by subtracting the rate for civilians from the rate for veterans or active duty personnel.

^c^ *P* value of difference in rates of veterans or active duty personnel and civilians, obtained through an *F* test.

Table 2. Frequency of Cigarette Smoking Among Adults With and Without Coronary Heart Disease by Sex, Age Group, and Veteran Status — United States, 2011–2012

| Disease Status Sex, Age Group (No. of observations), Smoking Frequency | Prevalence Rate, % (95% Confidence Interval) | | Difference Between Veterans/Active Duty Personnel and Civilians ^a^ (*P* Value)^b^ |
| --- | --- | --- | --- |
|  | Veterans/Active Duty Personnel | Civilians |  |
| **With Coronary Heart Disease** | | | |
| **Women** | | | |
| Aged 18–34 years (veterans or active duty personnel: 23; civilians: 581) | | | |
| Every day | 19.1 (0.0–42.0) | 23.2 (18.1–28.4) | −4.1 (.03) |
| Some days | 8.6 (0.0–20.0) | 5.8 (3.2–8.3) | 2.8 (.59) |
| Aged 35–44 years (veterans or active duty personnel: 32; civilians: 1,278) | | | |
| Every day | 31.6 (9.4–53.8) | 30.5 (25.9–35.1) | 1.1 (.22) |
| Some days | 12.1 (0.0–28.3) | 9.4 (5.9–12.8) | 2.7 (.74) |
| Aged 45–49 years (veterans or active duty personnel: 38; civilians: 1,425) | | | |
| Every day | 24.4 (3.2–45.6) | 33.4 (28.5–38.4) | −9.1 (.42) |
| Some days | 5.2 (0.0–12.3) | 9.6 (7.3–12.0) | −4.4 (.20) |
| Aged 50–54 years (veterans or active duty personnel: 79; civilians: 2,608) | | | |
| Every day | 30.5 (16.1–44.8) | 27.3 (24.2–30.5) | 3.1 (.04) |
| Some days | 16.3 (2.5–30.0) | 11.1 (8.8–13.4) | 5.2 (.69) |
| Aged 55–59 years (veterans or active duty personnel: 82; civilians: 3,869) | | | |
| Every day | 33.1 (14.8–51.4) | 22.5 (19.8–25.1) | 10.7 (.06) |
| Some days | 14.1 (3.4–24.8) | 11.5 (9.2–13.8) | 2.6 (.21) |
| Aged 60–64 years (veterans or active duty personnel: 96; civilians: 5,034) | | | |
| Every day | 14.9 (6.6–23.2) | 16.5 (14.6–18.5) | −1.6 (.64) |
| Some days | 13.1 (0.0–27.0) | 7.2 (5.9–8.6) | 5.9 (.71) |
| Aged 65–69 years (veterans or active duty personnel: 79; civilians: 5,610) | | | |
| Every day | 33.2 (14.9–51.4) | 12.9 (11.3–14.5) | 20.3 (.59) |
| Some days | 4.7 (0.0–10.7) | 5.4 (4.1–6.7) | −0.7 (.05) |
| Aged 70–74 years (veterans or active duty personnel: 98; civilians: 5,922) | | | |
| Every day | 9.5 (1.8–17.2) | 9.9 (7.4–12.4) | −0.4 (.05) |
| Some days | 5.4 (0.1–10.6) | 3.9 (2.9–4.9) | 1.5 (.70) |
| Aged 75–79 years (veterans or active duty personnel: 82; civilians: 6,024) | | | |
| Every day | 10.4 (1.7–19.1) | 5.0 (4.0–5.9) | 5.4 (.07) |
| Some days | 5.9 (0.0–14.1) | 2.4 (1.8–2.9) | 3.6 (.91) |
| Aged 80 years or older (veterans or active duty personnel: 166; civilians: 10,222) | | | |
| Every day | 3.8 (0.1–7.4) | 2.1 (1.7–2.5) | 1.7 (.71) |
| Some days | 0.5 (0.0–1.3) | 1.0 (0.7–1.2) | −0.4 (.29) |
| **Men** | | | |
| Aged 18–34 years (veterans or active duty personnel: 52; civilians: 506) | | | |
| Every day | 50.4 (28.1–72.6) | 23.8 (18.4–29.2) | 26.6 (.01) |
| Some days | 11.1 (0.0–22.4) | 13.0 (8.2–17.8) | −1.9 (.73) |
| Aged 35–44 years (veterans or active duty personnel: 187; civilians: 992) | | | |
| Every day | 28.7 (16.6–40.9) | 31.6 (26.7–36.6) | −2.9 (.31) |
| Some days | 22.7 (7.8–37.6) | 11.8 (8.3–15.3) | 10.9 (.85) |
| Aged 45–49 years (veterans or active duty personnel: 269; civilians: 1,166) | | | |
| Every day | 29.7 (18.7–40.7) | 28.3 (23.1–33.6) | 1.4 (.01) |
| Some days | 9.2 (3.5–14.9) | 12.4 (9.0–15.9) | −3.3 (.01) |
| Aged 50–54 years (veterans or active duty personnel: 587; civilians: 2,207) | | | |
| Every day | 33.9 (26.9–40.9) | 24.6 (21.3–27.9) | 9.3 (<.001) |
| Some days | 8.9 (4.3–13.5) | 10.7 (7.6–13.8) | −1.8 (.17) |
| Aged 55–59 years (veterans or active duty personnel: 1,160; civilians: 3,423) | | | |
| Every day | 28.6 (24.2–33.0) | 20.5 (17.9–23.0) | 8.1 (<.001) |
| Some days | 11.2 (7.9–14.4) | 9.5 (7.5–11.5) | 1.7 (.004) |
| Aged 60–64 years (veterans or active duty personnel: 2,888; civilians: 3,745) | | | |
| Every day | 18.2 (15.8–20.6) | 15.9 (13.6–18.1) | 2.3 (<.001) |
| Some days | 6.6 (5.0–8.3) | 5.6 (4.0–7.2) | 1.0 (.15) |
| Aged 65–69 years (veterans or active duty personnel: 3,623; civilians: 3,733) | | | |
| Every day | 14.3 (12.2–16.3) | 11.7 (9.9–13.5) | 2.6 (.04) |
| Some days | 3.9 (3.0–4.8) | 4.4 (3.3–5.4) | −0.5 (.25) |
| Aged 70–74 years (veterans or active duty personnel: 3,462; civilians: 3,223 | | | |
| Every day | 8.5 (7.0–10.1) | 7.2 (5.8–8.7) | 1.3 (.13) |
| Some days | 2.9 (1.9–3.9) | 2.5 (1.1–3.9) | 0.4 (.81) |
| Aged 75–79 years (veterans or active duty personnel: 3,756; civilians: 2,056) | | | |
| Every day | 6.9 (5.1–8.8) | 4.6 (3.1–6.1) | 2.3 (.45) |
| Some days | 1.1 (0.7–1.6) | 1.9 (1.0–2.9) | −0.8 (.97) |
| Aged 80 years or older (veterans or active duty personnel: 5,857; civilians: 1,773) | | | |
| Every day | 2.1 (1.5–2.6) | 1.3 (0.7–1.9) | 0.8 (.16) |
| Some days | 0.5 (0.3–0.8) | 0.7 (0.0–1.4) | −0.2 (.24) |
| **Without Coronary Heart Disease** | | | |
| **Women** | | | |
| Aged 18–34 years (veterans or active duty personnel: 1,445; civilians: 77,460) | | | |
| Every day | 17.5 (14.1–21.0) | 13.6 (13.2–14.0) | 3.9 (.03) |
| Some days | 5.6 (3.9–7.3) | 6.2 (5.8–6.5) | −0.5 (.59) |
| Aged 35–44 years (veterans or active duty personnel: 1,617; civilians: 69,438) | | | |
| Every day | 15.7 (12.2–19.2) | 13.4 (13.0–13.9) | 2.3 (.22) |
| Some days | 4.7 (3.2–6.2) | 4.9 (4.6–5.2) | −0.3 (.74) |
| Aged 45–49 years (veterans or active duty personnel: 1,016; civilians: 42,141) | | | |
| Every day | 14.4 (11.2–17.5) | 15.5 (14.8–16.1) | −1.1 (.42) |
| Some days | 7.3 (3.9–10.7) | 4.8 (4.4–5.2) | 2.5 (.20) |
| Aged 50–54 years (veterans or active duty personnel: 1,247; civilians: 53,115) | | | |
| Every day | 19.0 (15.5–22.6) | 15.3 (14.7–15.9) | 3.7 (.04) |
| Some days | 4.6 (2.8–6.5) | 5.2 (4.9–5.6) | −0.6 (.69) |
| Aged 55–59 years (veterans or active duty personnel: 1,104; civilians: 56,436) | | | |
| Every day | 15.5 (11.7–19.4) | 12.4 (11.8–12.9) | 3.2 (.06) |
| Some days | 6.5 (3.6–9.5) | 4.7 (4.3–5.1) | 1.8 (.21) |
| Aged 60–64 years (veterans or active duty personnel: 876; civilians: 57,787) | | | |
| Every day | 9.0 (6.3–11.7) | 9.8 (9.3–10.2) | −0.8 (.64) |
| Some days | 3.9 (1.2–6.6) | 3.8 (3.5–4.1) | 0. 1 (.71) |
| Aged 65–69 years (veterans or active duty personnel: 577; civilians: 49,722) | | | |
| Every day | 6.7 (4.2–9.1) | 8.4 (7.9–8.8) | −1.7 (.59) |
| Some days | 2.1 (0.9–3.2) | 3.4 (3.1–3.8) | −1.4 (.05) |
| Aged 70–75 years (veterans or active duty personnel: 427; civilians: 40,130) | | | |
| Every day | 11.2 (6.5–15.9) | 6.2 (5.8–6.6) | 5.0 (.05) |
| Some days | 2.9 (0.0–6.0) | 2.6 (2.3–2.8) | 0.3 (.70) |
| Aged 75–79 years (veterans or active duty personnel: 349; civilians: 31,359) | | | |
| Every day | 9.7 (3.3–16.1) | 4.8 (4.4–5.3) | 4.9 (.07) |
| Some days | 1.1 (0.0–2.5) | 1.9 (1.7–2.2) | −0.8 (.91) |
| Aged 80 years or older (veterans or active duty personnel: 689; civilians: 42,692) | | | |
| Every day | 2.2 (0.9–3.5) | 2.2 (2.0–2.5) | −.002 (.71) |
| Some days | 2.3 (0.3–4.2) | 1.1 (0.9–1.3) | 1.2 (.29) |
| **Men** | | | |
| Aged 18–34 years (veterans or active duty personnel: 5,483; civilians: 57,363) | | | |
| Every day | 19.5 (17.8–21.2) | 17.2 (16.6–17.7) | 2.3 (.005) |
| Some days | 10.2 (8.9–11.5) | 9.9 (9.5–10.4) | 0.3 (.73) |
| Aged 35–44 years (veterans or active duty personnel: 6,615; civilians: 42,650) | | | |
| Every day | 16.5 (15.0–18.1) | 15.6 (14.9–16.2) | 0.9 (.31) |
| Some days | 6.5 (5.4–7.6) | 6.8 (6.4–7.3) | −0.3 (.85) |
| Aged 45–49 years (veterans or active duty personnel: 4,823; civilians: 25,358) | | | |
| Every day | 18.6 (16.7–20.6) | 15.8 (15.0–16.7) | 2.8 (.01) |
| Some days | 4.2 (3.2–5.1) | 5.6 (5.1–6.2) | −1.4 (.01) |
| Aged 50–54 years (veterans or active duty personnel: 5,779; civilians: 30,089) | | | |
| Every day | 22.5 (20.5–24.5) | 16.7 (15.9–17.5) | 5.8 (<.001) |
| Some days | 6.6 (5.3–7.9) | 5.5 (5.0–6.0) | 1.1 (.17) |
| Aged 55–59 years (veterans or active duty personnel: 6,817; civilians: 30,725) | | | |
| Every day | 22.6 (20.7–24.5) | 14.4 (13.6–15.2) | 8.2 (<.001) |
| Some days | 7.2 (6.0–8.4) | 5.1 (4.6–5.6) | 2.1 (.004) |
| Aged 60–64 years (veterans or active duty personnel: 13,605; civilians: 22,977) | | | |
| Every day | 15.9 (14.7–17.0) | 11.0 (10.2–11.7) | 4.9 (<.001) |
| Some days | 4.3 (3.6–5.0) | 3.9 (3.3–4.4) | 0.4 (.15) |
| Aged 65–69 years (veterans or active duty personnel: 13,946; civilians: 15,559) | | | |
| Every day | 11.6 (10.6–12.6) | 9.9 (8.9–10.9) | 1.7 (.004) |
| Some days | 2.7 (2.3–3.2) | 3.0 (2.5–3.6) | −0.3 (.25) |
| Aged 70–74 years (veterans or active duty personnel: 10,275; civilians: 10,890) | | | |
| Every day | 8.3 (7.4–9.3) | 7.5 (6.4–8.7) | 0.8 (.13) |
| Some days | 2.1 (1.7–2.5) | 2.1 (1.7–2.6) | −.008 (.81) |
| Aged 75–79 years (veterans or active duty personnel: 9,133; civilians: 5,625) | | | |
| Every day | 5.5 (4.6–6.3) | 5.7 (4.5–6.8) | −0.2 (.45) |
| Some days | 1.5 (1.0–2.1) | 1.2 (0.8–1.6) | 0.3 (.97) |
| Aged 80 years or older (veterans or active duty personnel: 12,419; civilians: 4,389) | | | |
| Every day | 2.9 (2.4–3.4) | 2.5 (1.7–3.3) | 0.4 (.16) |
| Some days | 0.9 (0.7–1.2) | 1.4 (0.5–2.3) | −0.4 (.24) |

Source: Behavioral Risk Factor Surveillance System, 2011 and 2012.

Note: Estimates were not age standardized within age group. Frequency of cigarette smoking is as reported for the previous 30 days.

^a^ Because of rounding, the difference presented in this column may differ from the result obtained by subtracting the rate for civilians from the rate for veterans/active duty personnel.

^b^ *P* value of difference in rates of veterans/active duty personnel and civilians, obtained through an *F* test.

Table 3. Attempt to Quit Smoking Cigarettes by Adult Current Smokers With and Those Without Coronary Heart Disease, by Age Group, Sex, and Veteran Status — United States, 2011–2012

| Disease Status | Veterans/Active Duty Personnel | | Civilians | | Difference Between Veteran/Active Duty Personnel and Civilians^a^ (*P* Value)^b^ |
| --- | --- | --- | --- | --- | --- |
|  | No. of Observations | Prevalence Rate, % (95% Confidence Interval) | No. of Observations | Prevalence Rate, % (95% Confidence Interval) |  |
| **With Coronary Heart Disease** | | | | | |
| **Women, age, y** | | | | | |
| 18−34 | 12 | 93.1 (84.0−100.0) | 197 | 68.1 (58.0–78.1) | 25.0 (.73) |
| 35−44 | 14 | 94.7 (86.7−100.0) | 499 | 67.2 (59.1−75.4) | 27.4 (.36) |
| 45−49 | 19 | 69.9 (33.2−100.0) | 608 | 68.5 (61.5−75.5) | 1.4 (.58) |
| 50−54 | 38 | 78.7 (59.4−98.1) | 988 | 71.2 (66.4−76.1) | 7.5 (.71) |
| 55−59 | 28 | 62.0 (30.3−93.7) | 1,233 | 73.3 (68.4−78.2) | −11.3 (.27) |
| 60−64 | 29 | 68.3 (43.9−92.6) | 1,193 | 66.9 (61.6−72.2) | 1.4 (.74) |
| 65−69 | 20 | 43.8 (12.6−75.1) | 1,073 | 66.0 (60.7−71.3) | −22.2 (.34) |
| 70−74 | 21 | 53.6 (21.2−85.9) | 798 | 65.0 (56.0−74.0) | −11.4 (.50) |
| 75−79 | 11 | 42.1 (4.2−80.1) | 486 | 65.0 (57.7−72.4) | −22.9 (.22) |
| ≥80 | 8 | 20.9 (0.0−48.2) | 361 | 52.9 (44.6−61.3) | −32.0 (.71) |
| **Men, age, y** | | | | | |
| 18−34 | 33 | 62.4 (36.2−88.5) | 211 | 68.4 (58.7−78.0) | −6.0 (.32) |
| 35−44 | 73 | 66.5 (46.4−86.6) | 425 | 63.9 (55.8−71.9) | 2.6 (.12) |
| 45−49 | 113 | 60.0 (39.2−80.7) | 444 | 68.0 (58.6−77.4) | −8.0 (.55) |
| 50−54 | 244 | 64.4 (53.4−75.4) | 745 | 65.4 (58.6−72.2) | −1.0 (.32) |
| 55−59 | 447 | 58.9 (51.2−66.6) | 935 | 62.6 (56.9−68.3) | −3.7 (.60) |
| 60−64 | 740 | 59.5 (53.6−65.5) | 794 | 61.0 (54.2−67.7) | 1.4 (.99) |
| 65−69 | 682 | 50.8 (44.3−57.3) | 607 | 55.4 (48.5−62.3) | −4.6 (.07) |
| 70−74 | 434 | 49.3 (40.9−57.7) | 342 | 56.2 (46.1−66.3) | −6.9 (.82) |
| 75−79 | 256 | 53.5 (41.5−65.6) | 143 | 47.8 (34.6−61.0) | 5.7 (.16) |
| ≥80 | 197 | 42.0 (30.3−53.7) | 50 | 45.0 (22.6−67.5) | −3.0 (.31) |
| **Without Coronary Heart Disease** | | | | | |
| **Women, age, y** | | | | | |
| 18−34 | 340 | 65.6 (57.0−74.2) | 15,771 | 64.9 (63.6−66.2) | 0.7 (.73) |
| 35−44 | 340 | 55.8 (45.5−66.0) | 12,343 | 61.5 (60.0−63.1) | −5.7 (.36) |
| 45−49 | 251 | 53.0 (42.3−63.8) | 8,515 | 55.9 (53.9−57.9) | −2.9 (.58) |
| 50−54 | 301 | 55.7 (46.6−64.7) | 10,922 | 58.0 (56.3−59.7) | −2.3 (.71) |
| 55−59 | 250 | 64.6 (54.7−74.6) | 9,455 | 57.0 (54.9−59.2) | 7.6 (.27) |
| 60−64 | 139 | 57.9 (43.7−72.1) | 8,097 | 55.0 (53.0−57.1) | 2.9 (.74) |
| 65−69 | 85 | 52.2 (37.1−67.2) | 6,066 | 54.8 (52.4−57.2) | −2.6 (.34) |
| 70−74 | 52 | 47.1 (26.0−68.1) | 3,806 | 52.0 (49.1−55.0) | −5.0 (.50) |
| 75−79 | 35 | 39.0 (10.8−67.2) | 2,144 | 50.7 (46.7−54.6) | −11.7 (.22) |
| ≥80 | 30 | 49.4 (22.8−75.9) | 1,493 | 47.8 (43.2−52.4) | 1.6 (.71) |
| **Men, age, y** | | | | | |
| 18−34 | 1,651 | 65.0 (61.3−68.7) | 14,739 | 66.9 (65.6−68.2) | −1.9 (.32) |
| 35−44 | 1,505 | 61.5 (57.4−65.6) | 8,525 | 58.0 (56.1−59.9) | 3.5 (.12) |
| 45−49 | 1,104 | 51.5 (46.4−56.6) | 5,111 | 52.4 (49.9−54.9) | −0.9 (.55) |
| 50−54 | 1,701 | 49.8 (45.2−54.3) | 6,198 | 53.1 (50.7−55.4) | −3.3 (.32) |
| 55−59 | 1,870 | 49.5 (45.1−53.8) | 5,607 | 50.4 (47.0−52.9) | −0.9 (.60) |
| 60−64 | 2,602 | 51.4 (47.9−54.9) | 3,321 | 50.9 (47.6−54.2) | 0.5 (.99) |
| 65−69 | 1,977 | 45.1 (41.0−49.2) | 1,878 | 49.9 (45.2−54.6) | −4.8 (.07) |
| 70−74 | 1,153 | 45.5 (40.4–50.7) | 1,088 | 44.5 (38.1−50.9) | 1.1 (.82) |
| 75−79 | 615 | 39.0 (31.8−46.2) | 402 | 52.9 (43.9−62.0) | −13.9 (.16) |
| ≥80 | 498 | 40.0 (32.5−47.5) | 170 | 48.5 (32.3−64.7) | −8.5 (.31) |

Source: Behavioral Risk Factor Surveillance System, 2011 and 2012.

Note: Estimates are not age standardized within age group. Current smokers who reported stopping smoking for at least 1 day in an attempt to quit during the previous 12 months were defined as having a quit attempt.

^a^ Because of rounding, the difference presented in this column may differ from the result obtained by subtracting the rate for civilians from the rate for veterans/active duty personnel.

^b^ *P* value of difference in rates of veterans/active duty personnel and civilians, obtained through an *F* test.
